# Hybrid Zinc Phthalocyanine/PVDF-HFP System for Reducing Biofouling in Water Desalination: DFT Theoretical and MolDock Investigations

**DOI:** 10.3390/polym16121738

**Published:** 2024-06-19

**Authors:** Bassem Jamoussi, Mohhamed Naif M. Al-Sharif, Lassaad Gzara, Hussam Organji, Talal B. Almeelbi, Radhouane Chakroun, Bandar A. Al-Mur, Naief H. M. Al Makishah, Mohamed H. F. Madkour, Fahed A. Aloufi, Riyadh F. Halawani

**Affiliations:** 1 Department of Environment, Faculty of Environmental Sciences, King Abdulaziz University, Jeddah 21589, Saudi Arabia; malsharif0134@stu.kau.edu.sa (M.N.M.A.-S.); talmeelbi@kau.edu.sa (T.B.A.); rshagroon@kau.edu.sa (R.C.); balmur@kau.edu.sa (B.A.A.-M.); nalmakishah@kau.edu.sa (N.H.M.A.M.); mmadkour@kau.edu.sa (M.H.F.M.); faloufi@kau.edu.sa (F.A.A.); rhalawani@kau.edu.sa (R.F.H.); 2 Center of Excellence in Desalination Technology, King Abdulaziz University, Jeddah 21589, Saudi Arabia; lgzara@kau.edu.sa (L.G.); haorganji@kau.edu.sa (H.O.)

**Keywords:** PVDF-HFP, Zn(4-PPOx) _4_Pc/PVDF-HFP, electrospinning, DFT, Molecular docking, biofouling, *S. aureus* (1N67)

## Abstract

Fouling and biofouling remain significant challenges in seawater desalination plants. One practical approach to address these issues is to develop anti-biofouling membranes. Therefore, novel hybrid zinc phthalocyanine/polyvinylidene fluoride-co-hexafluoropropylene (Zn(4-PPOx)_4_Pc/PVDF-HFP) membranes were prepared by electrospinning to evaluate their properties against biofouling. The hybrid nanofiber membrane was characterized by atomic force microscopy (AFM), attenuated total reflectance-Fourier transform infrared (ATR-FTIR) spectroscopy, and contact angle measurements. The theoretical calculations of PVDF-HFP, Zn(4-PPOx)_4_Pc), and Zn(4-PPOx)_4_Pc/PVDF-HFP nanofibers were performed using a hybrid functional RB3LYP and the 6-31 G (d,p) basis set, employing Gaussian 09. DFT calculations illustrated that the calculated physical and electronic parameters ensured the feasibility of the interaction of PVDF-HFP with Zn(4-PPOx)_4_Pc via a halogen–hydrogen bond, resulting in a highly stable and remarkably reactive structure. Moreover, molecular electrostatic potential (MEP) maps were drawn to identify the reactive regions of the Zn(4-PPOx)4Pc and PVDF-HFP/Zn(4-PPOx)4Pc nanofibers. Molecular docking analysis revealed that Zn(4-PPOx)_4_Pc has highest binding affinity (−8.56 kcal/mol) with protein from *S. aureus* (1N67) mainly with ten amino acids (ASP405, LYS374, GLU446, ASN406, ALA441, TYR372, LYS371, TYR448, LYS374, and ALA442). These findings highlight the promising potential of Zn(4-PPOx) _4_Pc/PVDF-HFP nanocomposite membranes in improving the efficiency of water desalination by reducing biofouling and providing antibacterial properties.

## 1. Introduction

Desalination technology has emerged as a critical solution to mitigate the rising global demand for freshwater, which is driven by factors such as population growth, industrialization, and climate change [1]. One of the major obstacles in desalination is the significant degradation of membrane processes along with water flux decline, known as biofouling, which significantly affects the operational costs and sustainability of desalination systems [2]. Biofouling is a process that occurs in multiple stages, including the attachment of microorganisms to the surface of the membrane, their proliferation, the formation of colonies, the accumulation of organic matter, and ultimately, the development of biofilms [3]. The growth of biofouling is related to the morphology of the membrane and the biological composition of the microorganism species at the interface, which involves a variety of physical and chemical processes, including adsorption, desorption, and diffusion [4]. Consequently, addressing biofouling issues is important for ensuring and improving the long-term efficiency and functionality of these membranes in water treatment, desalination, and filtration processes. Researchers have continually explored innovative strategies and technologies to mitigate biofouling effects and extend the lifespan of nanofiber membranes.

Over the past few decades, researchers have focused on preventing biofouling by modifying the membrane surfaces with nanoparticles and developing fouling-resistant membrane materials [5]. Furthermore, certain nanoparticles, such as titanium oxide (TiO_2_), zinc oxide (ZnO), copper (Cu), and silver (Ag), have been recognized as effective antibacterial agents against a wide range of microorganisms, including viruses, yeasts, fungi, and bacteria [5]. The attachment of microorganisms to membrane surfaces is influenced by numerous factors that can be categorized into three primary groups: properties of the membrane surface (e.g., roughness of the membrane surface, wetting characteristics, hydrophobicity, membrane surface charge, and pore dimensions), parameters of the source water (including temperature, pH, ionic strength, nutrient content, osmotic pressure, and velocity), and intrinsic properties of microorganisms (size, wetting characteristics of cell surfaces, and charge) [6,7,8]. To combat fouling, efforts have primarily focused on developing antifouling membranes with a specific emphasis on mitigating fouling caused by organic substances and biofoulants [9]. Antifouling technologies typically employ low-toxicity or nontoxic antifouling agents to eliminate microorganisms and achieve antifouling efficacy [10,11,12]. A study on the bacterial adhesion and fouling of reverse osmosis membranes was conducted by Ridgway et al., which demonstrated that hydrophobic interactions between bacterial cell surface components and the cellulose acetate membrane surface significantly contributed to the initial stages of bacterial adhesion and biofilm formation [8]. However, relying on a single antifouling agent may not yield optimal results in terms of its effect on diverse microorganisms and could potentially accumulate on membrane surfaces. Additionally, prolonged use of antifouling agents can engender microbial resistance, gradually diminishing their antifouling efficacy over time.

To address these limitations, electrospinning has been recognized as an innovative and versatile method for producing nanofibers. These nanofibers are highly sought after because of their exceptional filtration and water treatment capabilities owing to their high efficiency, fine pore structure, and excellent permeability, all of which are achieved at a cost-effective rate. By incorporating various functional materials into electrospun nanofibers, we can develop composite membranes that offer enhanced and sustained antifouling properties, thereby overcoming the drawbacks associated with single-agent antifouling strategies [13]. These fibers, renowned for their nanoscale diameters, offer a vast surface-to-volume ratio and heightened porosity, enhancing their suitability for diverse applications through their potential for surface functionalization. Their tunable porosity contributes to a substantial specific surface area, numerous active sites, and improved material attributes, such as permeability and selectivity, while minimizing fouling, thereby highlighting their importance in advancing desalination and water purification technologies [14]. The fiber-stretching mechanism is influenced by several factors, including Coulomb repulsion, surface tension and solutions, viscoelasticity, polymer weight, and architecture [15]. Strategic alignment and patterning enable the formation of structured or hierarchical nanofiber configurations. The points of intersection in nonwoven mats can be strengthened to form fiber connections, with possibilities for physical or chemical adjustments to modify the porosity and pore size, and incorporate functional elements, further enabling the creation of three-dimensional structures through the vertical expansion of nanofiber layers [16]. Advancements in electrospinning have not only enhanced the production efficiency but also broadened the spectrum of available nanofiber morphologies. In this context, Albiladi et al. crafted hydrophobic PVDF-HFP nanofiber membranes specifically for brine treatment via membrane distillation. Their research delved into how solvent composition influences electrospinning and its effects on membrane characteristics and performance in brine purification [17].

Polyvinylidene fluoride (PVDF) differs from other polymers due to its exceptional properties, including mechanical strength, high dipole moment, high polarizability, good flexibility, and superior resilience, in addition to its higher actuation strain [18,19]. Building on these unique attributes, Li et al. successfully integrated fluorinated zinc oxide (ZnO) into an electrospun PVDF framework to create a nanofibrous membrane with superhydrophobic characteristics [20]. Furthermore, incorporating the hexafluoropropylene (HFP) group with polyvinylidene fluoride (PVDF) enhances PVDF-co-HFP with improved properties such as lower crystallinity, higher solubility, stronger mechanical properties, greater free volume, and increased hydrophobicity. These enhancements underscore why PVDF-co-HFP has received significant attention as a promising membrane material, particularly in advanced applications such as water treatment and desalination, demonstrating the profound impact of electrospinning and material innovation in this field [21,22].

Research has been conducted extensively on the membrane composites created through the functionalization of nanofibers to develop innovative, efficient, and biofouling-resistant membranes. Functionalization agents may be added to the spinning solution before electrospinning to pre-functionalize nanofibers. In addition to conventional functionalization agents, phthalocyanines can be used as novel and innovative agents [23,24]. This approach allows for the integration of unique properties into the nanofibers, further enhancing their performance and expanding their applications in various filtration and water purification technologies. Studies have shown that phthalocyanines effectively inhibit various Gram-negative and Gram-positive bacteria, making them promising candidates for use in bacterial-resistant nanofiber membranes [25]. Furthermore, the immobilization of phthalocyanines on electrospun nanofiber membranes extends their applications beyond filtration and desalination. Recent advances in electrospun nanofiber composites incorporating phthalocyanines have demonstrated their utility in environmental monitoring, such as the use of electrospun PVC-nickel phthalocyanine composites as microsensors to measure methanol vapor [26], as well as optoelectronic sensors exhibiting unique properties of photoluminescence, using electrospun nanofibers of poly(ethylene oxide)/copper(II) tetraamino-phthalocyanine hybrids [27]. Thus, the use of phthalocyanines as functionalizing agents opens up new possibilities and perspectives in the field of composite membranes.

In this study, we investigated whether zinc 2,9,16,23-tetra(4-propylphenoxy) phthalocyanine (Zn(4-PPOx)_4_Pc) can be incorporated into uniform PVDF-HFP nanofiber membranes prepared by electrospinning and evaluate their potential to enhance anti-biofouling properties against Gram-positive and Gram-negative bacteria. This study focused on the development of a novel hybrid membrane system that combines zinc phthalocyanine 2,9,16,23-tetra(4-propylphenoxy) (Zn(4-PPOx)_4_Pc) with polyvinylidene fluoride- co-hexafluoropropylene (PVDF-HFP) by electrospinning, a stage of synthesis of Zn(4-PPOx)_4_Pc involving a series of chemical reactions to ensure the desired functionalization, followed by its incorporation into the PVDF-HFP matrix by electrospinning. The Zn(4-PPOx)_4_Pc/PVDF-HFP membrane was characterized by structural, chemical, and morphological analyses. In addition to the physical characterization, the wettability and surface energy of the membranes were evaluated using contact angle measurements with various mixtures of water and ethanol. Density functional theory (DFT) calculations were carried out in order to better understand the electronic properties and the potential interactions between PVDF and HFP and Zn(4-PPOx)_4_Pc, these interactions were used to determine the stability and the membrane reactivity. To understand the antimicrobial potential of the hybrid membranes, molecular docking was used to explore the binding affinities of the membranes with bacterial proteins. Molecular docking simulations and DFT calculations can effectively prevent biofouling in desalination applications.

## 2. Materials and Methods

### 2.1. Materials

Poly (vinylidene fluoride-co-hexafluoropropylene) PVDF-HFP Solef^®^21216 (Solvay Specialty Polymers, Bollate, Italy; Mw: 600,000 g·mol^−1^) was dried for a few minutes at nearly 60 °C before use to remove any moisture. 4-Nitrophthalonitrile was prepared and purified according to the methods described in our previous research [28,29]. N,N-dimethylaminoethanol (DMAE, ≥99.5% purity), N, N-Dimethylformamide (DMF, ≥99.9% purity), 1,8-diazabicyclo [5.4.0]undec-7-ene (DBU, ≥99.0% purity), 4-propylphenol (99% purity) and zinc acetate dihydrate (≥99.0% purity) were purchased from Sigma-Aldrich (Steinheim, Germany). Nitric acid (HNO_3_, >90% purity)), ethyl ether (C_4_H_10_O, 95%), and anhydrous sodium sulfate (Na_2_SO_4_, 99% purity) were supplied by Merck KGaA (Darmstadt, Germany).

### 2.2. Instruments

The characterization of phthalonitrile and phthalocyanine was carried out using ^1^H NMR and ^13^C NMR (Agilent Technologies VNMRS 500 MHz, Lexington, MA, USA) as well as a MALDI-TOF/MS system, the MALDI Biotyper system (microflex LT; Bruker Daltonik GmbH, Bremen, Germany). The absorption spectra were measured using a Shimadzu UV-Vis spectrometer with a resolution of 0.1 nm. The surface morphology and roughness of the membranes were characterized using atomic force microscopy (AFM) on the AFM XE-7-Park system. The membrane surface hydrophobicity was assessed by measuring the water contact angle of each membrane using a Theta optical tensiometer. The surface chemical compositions and functional groups of all membranes were analyzed using Cary 360 Fourier-transform infrared spectroscopy (FTIR) and all FTIR spectra were recorded using the attenuated total reflection (ATR) technique in the wavelength range of 4000–400 cm^−1^.

### 2.3. Synthesis of 4-(4-propylphenoxy)phthalonitrile

**Figure 1:**
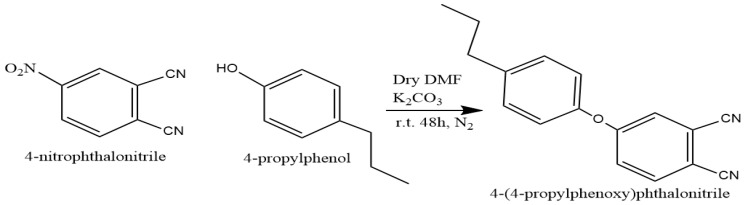
Syntesis of 4-(4-propylphenoxy) phthalonitrile.

4-(4-propylphenoxy) phthalonitrile was prepared through a base-catalyzed nucleophilic aromatic nitro displacement reaction. This process involves the combination of 4-nitrophthalonitrile and 4-propylphenol, with K_2_CO_3_ serving as the base in anhydrous DMF, as depicted in the reaction scheme in Figure 1.

To synthesize 4-(4-propylphenoxy) phthalonitrile, 4-Nitrophthalonitrile (2.5 g, 14.5 mmol) was dissolved in dry N, N-dimethylformamide (60 mL), and 4-propylphenol (4.9 g, 36.1 mmol) was added. After stirring for 15 min, anhydrous potassium carbonate (7.5 g, 54 mmol) was added in small portions over a period of two hours with efficient stirring. The reaction mixture was then stirred magnetically at room temperature for 48 h. After the reaction was deemed complete by means of thin-layer chromatography, the mixture was poured into a mixture of ice and water (400 mL), and the precipitate was filtered and washed with water until the filtrate was neutral. The precipitate was then dried under reduced pressure, and finally, it was crystallized from ethanol to yield 4-(4-propylphenoxy)phthalonitrile.

Yield: 75%; ^1^H NMR (500 MHz, DMSO-*d*6), δ 8.16 (d, ^1^H), 7.94 (s, ^1^H), 7.58 (d, ^1^H), 7.33(dd, ^2^H), 7.09 (dd, ^2^H), 2.61 (m, ^2^H), 1.64 (m,^2^H), 0.94 (m,^3^H) ppm. ^13^C NMR (126 MHz, acetone-*d*6), δ 162.0, 154.30, 115.5, 108.90, 135.10, 121.80, 128.5, 122.5, 121.8, 134.4, 128.9,115.8,37.9, 24.1,13.7 ppm. FT-IR υ_max_ (cm^−1^): 3080 (Ar-CH), 2238 (C≡N), 1245 (O-C), MALDI-TOF, (*m*/*z*) calcd.: 262.11; found: 264.12 [M + H]^+^.

### 2.4. Synthesis of Zinc 2,9,16,23-tetra(4-propylphenoxy) Phthalocyanine (Zn(4-PPOx)_4_Pc)

**Figure 2:**
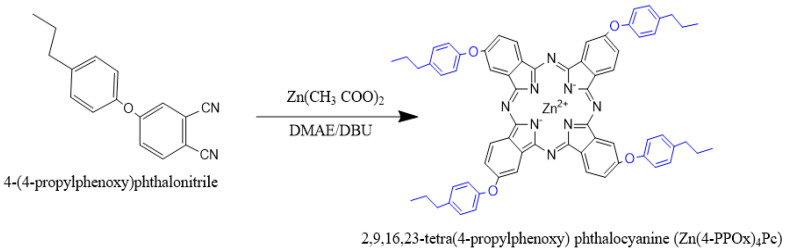
Synthesis of zinc 2,9,16,23-tetra(4-propylphenoxy) phthalocyanine.

Zinc 2,9,16,23-tetra(4-propylphenoxy) phthalocyanine (Zn(4-PPOx)_4_Pc) was synthesized according to the reaction scheme shown in Figure 2.

To synthesize Zn(4-PPOx)4Pc, 2 g (7.62 mmol) of 4-(4-propylphenoxy) phthalonitrile and 0.42 g (1.96 mmol) of zinc acetate were dissolved in 60 mL of dimethylaminoethanol in a nitrogen-purged reflux apparatus. The temperature was increased to 90 °C, and ten drops of DBU were added to the reaction mixture. The temperature was then increased to 150 °C, and the mixture was stirred for 48 h under N2, with progress monitored by TLC. After cooling, the mixture was diluted with ethyl alcohol, and the resulting precipitate was filtered off. The crude product was treated with diethyl ether, dichloromethane, and hexane before being dried under reduced pressure. The product was further purified using column chromatography on silica gel with CHCl_3_ as the eluent. Yield: 58%; ^1^H NMR (500 MHz, DMSO-d6), 7.09–7.33 (m, 28H), 2.61(t,8H), 1.64(m,8H), 0.94(t, 12H) ppm. [ATR vmax/cm^−l^]: 3033–3013 (Ar-CH), 3080 (Ar-CH), 2238 (C=N), 1480 (C=C), 1100–1302 (O-C), 977, 865, 789, 733 (C-C); UV-Vis (DMF): λmax/nm: 347, 610, 676, Calc. for C68H56N8O4Zn:C 77.84; H, 5.38; N 10.68; O 6.10%; Found C77.72, H 5.31, N 10.64, O 6.03%. Mass spectra-MS m/z:1114.63; found 1114.15 [M + H]+.

### 2.5. Preparation of Phthalocyanine-PVDF and PVDF-HFP Nanocomposite Membranes by Electrospinning

#### 2.5.1. Process of the Preparation of Polymer Solution

Based on the research conducted by Gzara et al. [30], it is recommended that the solvent ratio be set at 6:4 (DMF/acetone) and the percentage of PVDF-HFP be 12% in order to improve the hydrophobicity of nanofibrous PVDF-HFP membranes through the use of the electrospinning technique. These ratios were utilized in our subsequent work in preparing composite membranes. The same method was used to fabricate the control and composite membranes. The electrospinning solution contained a mixture of 12.2 mg of phthalocyanine molecules, 6 g of PVDP-HFP 21216, and 44 g of a mixture of DMF and acetone (6:4).

#### 2.5.2. Fabrication of Zn(4-PPOx)_4_Pc/PVDF-HFP Nanofibers by Electrospinning Technique

**Figure 3:**
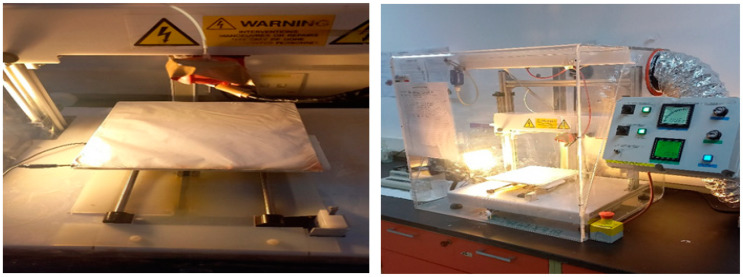
Membrane manufacturing process by the electrospinning device.

The production of the nanofibrous Zn(4-PPOx)_4_Pc/PVDF-HFP material was carried out through the use of electrospinning, which involved the application of a 12% polymer solution with a DMF/acetone weight ratio of 6:4. This solution was transferred into a 10 mL plastic syringe and connected to a syringe pump with a feed rate of 1 mL/h. The positive electrode of a high-voltage power source was attached to the metal needle tips, which were connected to the syringe. The applied voltage was 20 kV, and the distance from the tip of the syringe to the collector was 17 cm. The needle and collector plate were manipulated in two dimensions to achieve a uniform surface thickness of the collected material. The electrospinning machine used in this study was sourced from DeltaE SRL in Italy, and a visual representation of the apparatus can be found in Figure 3. After production, every electrospun membrane undergoes a rigorous heat treatment process to ensure its integrity. This process begins with heating the membrane to 50 °C overnight to remove any residual solvent. A post-heat treatment procedure is then carried out, which involves sandwiching the electrospun membrane between two glass plates and subjecting it to six hours of pressurization at 130 °C. This process fuses the nanofibers together, enhancing the structural integrity of the membrane.

### 2.6. Computational Methodology

Density Functional Theory (DFT) calculations were performed using Gaussian 09 software (Revision A02; Gaussian, Inc.: Wallingford, CT, USA, 2009) hosted on the Aziz supercomputing platform at the High-Performance Computing Centre of King Abdulaziz University (http://hpc.kau.edu.sa; accessed on 1 May 2024). Geometry optimization of PVDF-HFP, zinc tetra(4-propylphenoxy) phthalocyanine (Zn(4-PPOx)_4_Pc), and their interactions was performed using the hybrid functional B3LYP method [31], which incorporates Becke’s three-parameter exchange functional, along with the Lee-Yang-Parr correlation functional. The basis sets utilized include 6-31G+(d,p) for geometry optimization for calculating the corresponding harmonic vibration frequencies. In this study, zinc (Zn) metal was coordinated with phthalocyanine (Pc) in a divalent (2+) oxidation state. The valence pseudo-orbitals were refined using B3LYP hybrid exchange-correlation functionals. The exponents for the primitive Gaussian functions were maintained at their initially optimized values, and the contraction scheme remained unchanged. However, the contraction coefficients underwent systematic optimization. This involved sequential self-consistent field (SCF) optimizations for each contraction coefficient, which sequentially informed the input estimates for Gaussian09 software (Revision A02; Gaussian, Inc.: Wallingford, CT, USA, 2009). All calculations were performed in the gas phase at a temperature of 298 K and standard atmospheric pressure.

**Figure 4:**
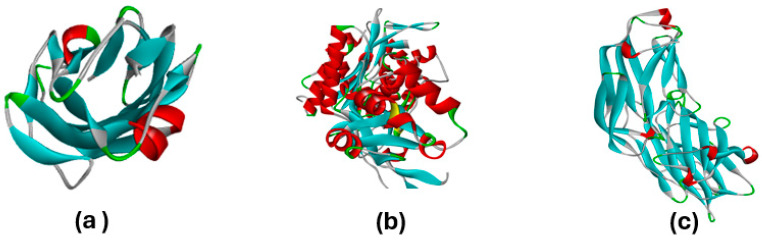
Crystal structures of bacterial proteins from *E. coli* (PDB ID: 4XO8) (**a**), *S. aureus* (PDB ID: 1N67) (**b**), and *P. aeruginosa* (PDB ID:3PBQ) (**c**).

The crystal structures of bacterial proteins from *E. coli* (PDB ID: 4XO8), *S. aureus* (PDB ID: 1N67), and *P. aeruginosa* (PDB ID:3PBQ) [32] (Figure 4) were obtained in PDB format from the Protein Data Bank [33,34]. The interactions of Zn(4-PPOx)_4_Pc with these bacterial proteins were zanalyzed using the molecular modeling software AutoDock 4.2 (version 4.2.6, released in 2009) [35,36]. The docking analysis utilizing the AutoDockTools (ADT/AutoDockTools -AutoDock (https://autodocksuite.scripps.edu/adt/) accessed on 1 May 2024) suite was used to prepare the ligand and receptor structures, assigning Gasteiger charges and adjusting ligand rotatable bonds [37]. Following the docking process, both macromolecules and ligands were saved in pdbqt format and converted to pdb format as required by the Discovery Studio visualizer screening tool. This tool was used to examine and visualize the docking results and investigate the nonbonding interactions between the ligands and amino acid residues of the receptor protein. Docking configurations and binding energies were determined using the Lamarckian genetic algorithm.

#### Electrostatic Surface Potential Method

Electrostatic potential maps are vital tools for visualizing the charge distribution of molecules, which in turn can predict the reactivity and molecular interactions [38]. These maps illustrate how a molecule may attract or repel charged entities, with negatively charged regions being susceptible to positively charged species such as protons, and vice versa. The electrostatic potential energy reflects the interaction strength between charges at specific locations around the molecule [39] and can be obtained from DFT calculations for accurate mapping. Visual representations such as electrostatic potential maps zutilize a color gradient, often from red to blue, to denote the spectrum of potential values [40], thus making the data intuitively accessible. Red areas indicate lower potential energies (electron-rich regions), while blue areas indicate higher potential energies (electron-poor or positively charged regions). The resulting map overlaid on the molecule’s electron density surface shows the charge distribution of a molecule that envelopes the molecule, offering insights into its electronic structure and subsequent chemical behavior.

## 3. Results

### 3.1. Absorption Properties of Zn(4-PPOx)_4_Pc

**Figure 5:**
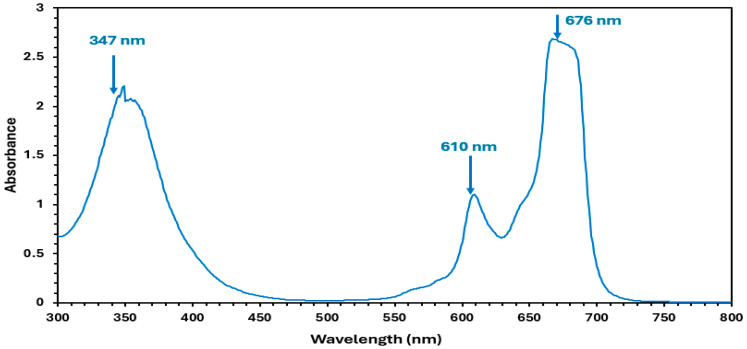
UV-Vis Spectrum of Zn(4-PPOx)_4_Pc.

The spectral absorption spectrum of Zn(4-PPOx) _4_Pc (Figure 5) exhibited two main absorption bonds that are distinct those from of phthalocyanines. The first of these bands is the B (or Soret) band (347 nm), which ranges from 300 to 400 nm, while the second is the Q band (676 nm), which is located between 600 and 750 nm. The B band results from the transition between the (a2u) and (eg) lowest unoccupied molecular orbitals (LUMO), whereas the Q band is primarily caused by charge transfer from the pyrrolic carbons to other atoms within the molecule. The visible region, encompassing the π–π* transition of the conjugated system, specifically the doubly degenerated transition (a1u-eg) from the highest occupied molecular orbital (HOMO) to the LUMO, is associated with the first band [41]. The splitting of the peaks in the Q-band region can be attributed to the presence of dimeric species in the solution [42]. These bands confirm the structure of Zn(4-PPOx)_4_Pc and are characteristic of phthalocyanines.

### 3.2. Characterizations of PVDF-HFP/Zn(4-PPOx)_4_Pc Nanofiber Membranes

#### 3.2.1. Surface Characterization by FTIR/ATR Spectroscopy

**Figure 6:**
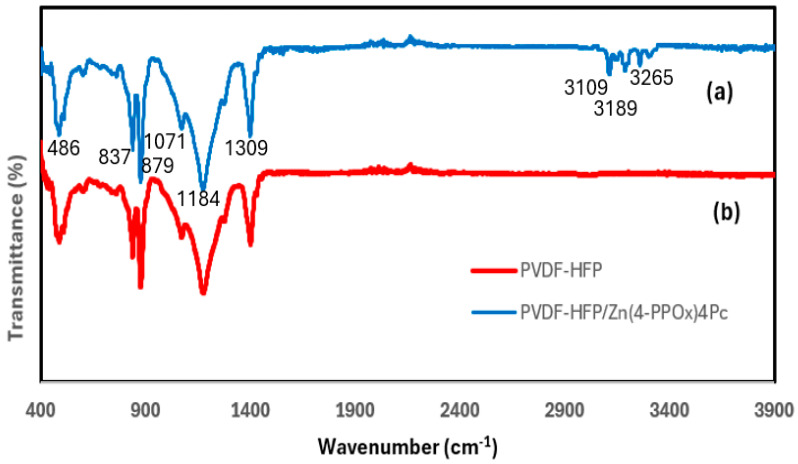
FTIR results of PVDF-HFP/Zn(4-PPOx)_4_Pc (**a**) and PVDF-HFP (**b**) membrane.

Figure 6 shows the FTIR results for the nanofibers, displaying various key peaks corresponding to the vibrational modes of the functional groups present in both membranes. The vibrational peak at 486 cm^−1^ is characteristic of CF_2_ bending vibrations. The bands observed at 837 cm^−1^ and 879 cm^−1^ are associated with out-of-plane deformation vibrations of CH_2_ groups and CF_2_ stretching vibrations, while its swinging vibration is shown at 1179 cm^−1^. The vibrational peak at 1274 cm^−1^ is assigned to the bending and asymmetric stretching vibrations of the CF_2_ group. The peak observed at 1399 cm^−1^ was related to the wagging vibration of CH_2_ or the stretching vibrations of the CF group. The vibrational peak at 1399 cm^−1^ is associated with the bending vibrations of the CH_2_ groups [43,44].

The lack of significant absorption in the 3000–3500 cm^−1^ region supports the hydrophobic nature of PVDF-HFP. All the aforementioned vibrational peaks were observed in the FTIR spectra of the PVDF-HFP/Zn(4-PPOx) _4_Pc nanofiber membranes. In the 2500–3000 cm^−1^ region, multiple peaks exist only in PVDF-HFP/Zn(4-PPOx) _4_Pc nanofiber membranes, which are attributed to the C-H stretching vibrations from the aliphatic chains of the 4-propylphenoxy substituents.

In the spectral range of 3100 to 4000 cm^−1^, the phthalocyanine core and phenoxy rings exhibit aromatic C-H stretching vibrations. However, these vibrations can be affected by F-H electrostatic interactions in the IR spectrum, causing N-H stretching vibrations to shift to lower frequencies (3100–3500 cm^−1^) and broaden the peaks. The presence of these bonds in the modified membrane suggests additional interactions or modifications resulting from the incorporation of Zn(4-PPOx)4Pc.

#### 3.2.2. Morphological Studies: AFM

**Figure 7:**
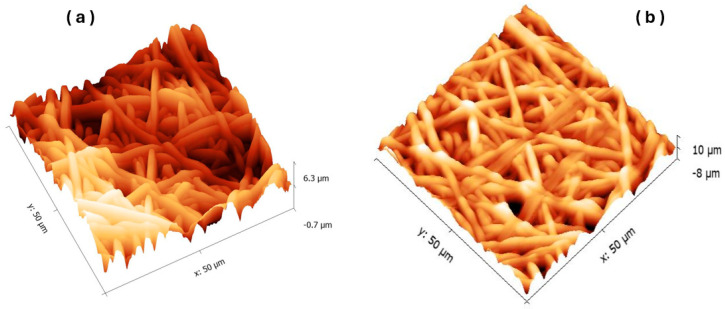
3D configuration of PVDF-HFP/Zn(4-PPOx)_4_Pc (**a**) and PVDF-HFP (**b**) membrane with a ratio of 50 µm.

The morphological characteristics of PVDF-HFP and PVDF-HFP/Zn(4-PPOx)_4_Pc nanofiber membranes were evaluated using the AFM technique. The 3D AFM images (Figure 7) were analyzed, and the parameters for the area roughness and line roughness of both types of nanofiber membranes are summarized in Table 1.

Upon comparing the Atomic Force Microscopy (AFM) results between samples PVDF-HFP/Zn(4-PPOx)_4_Pc and PVDF-HFP, it is evident that both samples exhibited comparable measured areas. However, the surface of sample PVDF-HFP was generally rougher than sample PVDF-HFP/Zn(4-PPOx)_4_Pc. This is demonstrated by the higher average roughness (Sa) and root mean square roughness (Sq) in the area roughness measurements for sample PVDF-HFP. Furthermore, in the line roughness measurements, sample PVDF-HFP displayed higher average roughness (Ra) and root mean square roughness (Rq), as well as greater maximum height (Ry), maximum peak height (Rp), and more negative maximum valley depth (Rv). The higher roughness of the PVDF-HFP membrane contributes to its higher contact angle, indicating greater hydrophobicity. In contrast, the incorporation of the Phthalocyanine into the PVDF-HFP matrix appeared to smoothen the membrane surface, as evidenced by the lower roughness values from AFM measurements. This smoother surface led to a reduction in the contact angle, thereby slightly hindering the inherent hydrophobic properties of the PVDF-HFP membrane. The reduced surface roughness of the PVDF-HFP/Zn(4-PPOx)_4_Pc membrane resulted in a more uniform and less hydrophobic surface than that of the unmodified PVDF-HFP membrane. This suggests that the presence of phthalocyanine modifies the surface morphology to decrease hydrophobicity, potentially enhancing the anti-biofouling performance of the composite membrane.

#### 3.2.3. Wettability and Surface Free Energy

**Figure 8:**
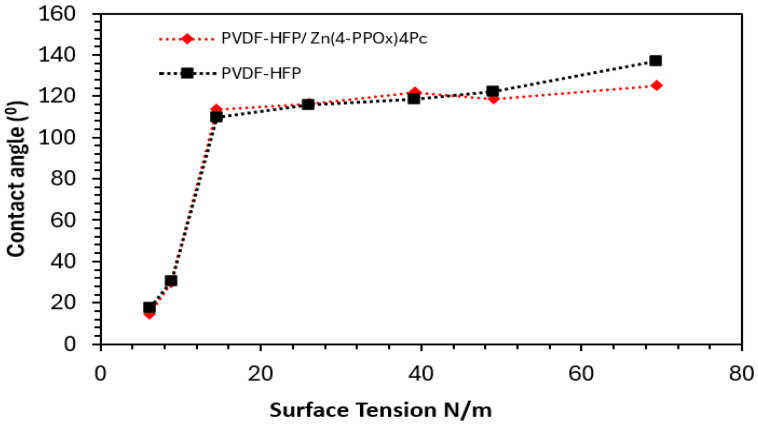
Contact angle and surface tension of the PVDF-HFP/Zn (4-PPOx)4Pc and PVDF-HFP membranes.

The interfacial interaction between the membrane and contaminants, and the resulting behaviors of adsorption, mass transfer, and membrane fouling during filtration, are closely linked^6,7^. The contact angle (CA) is extensively utilized to evaluate the hydrophilicity/hydrophobicity of a material’s surface, providing information on the surface’s wetting properties and surface free energy between the surface and the liquid. This enables the determination of solid surface energy parameters, such as the Lifshitz-van der Waals (γ_LW_) and Lewis acid/base components (γ+/γ−) of the membrane [45,46]. In this study, mixtures of water and ethanol at varying concentrations (100/0, 95/5, 90/10, 80/20, 70/30, 80/20, 90/10, 0/100) were used for contact angle measurements on both membranes. Figure 8 depicts the relationship between surface tension (N/m) and contact angle (°) for the PVDF-HFP membrane and the PVDF-HFP/Zn(4-PPOx)4Pc membrane.

**Figure 9:**
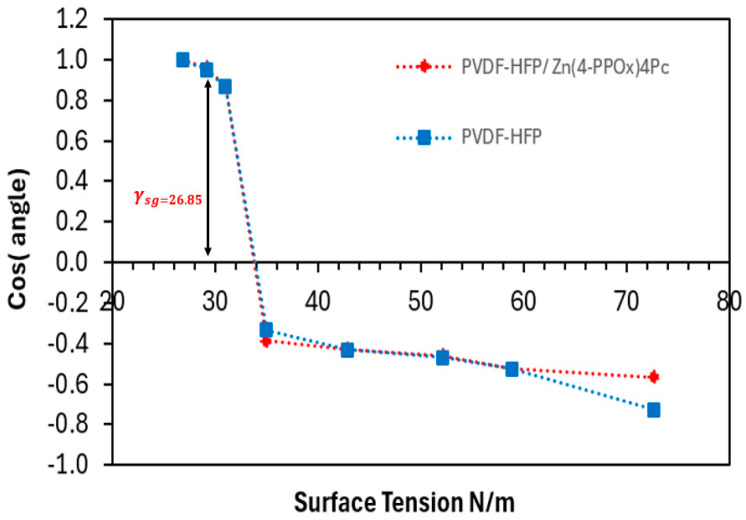
Cos (angle) and surface tension of PVDF-HFP/Zn (4-PPOx)_4_Pc and PVDF-HFP membranes.

Figure 8 illustrates that at a surface tension of 30 N/m, both membranes exhibited relatively high contact angles. Specifically, the PVDF-HFP membrane had an angle of approximately 20° ± 1.80, while the PVDF-HFP/Zn(4-PPOx)4Pc membrane displayed a slightly lower angle. This indicates that both membranes possess a more hydrophilic nature at lower surface tensions. As the surface tension rose from 30 to 50 N/m, there was a substantial increase in the contact angle for both membranes, reaching around 120° ± 3.36 at 50 N/m. This sharp rise suggests that the membranes become more hydrophobic with higher surface tension. Beyond 50 N/m, the contact angles for both membranes remained steady and showed minor fluctuations, suggesting a limit to the hydrophobicity enhancement achievable with further increases in surface tension. When comparing the two membranes, the PVDF-HFP membrane consistently displayed a slightly higher contact angle than the PVDF-HFP/Zn(4-PPOx)_4_Pc membrane over the range of surface tensions tested. This suggests that the addition of Phthalocyanine marginally reduces the hydrophobicity of the PVDF membrane. Although both membranes become more hydrophobic with increasing surface tension, the unmodified PVDF-HFP membrane maintains a higher level of hydrophobicity compared to the modified membrane. This difference could be attributed to the influence of Phthalocyanine, which appears to slightly hinder the inherent hydrophobic properties of the PVDF-HFP membrane. Figure 9 displays the cosine of the contact angle as a function of surface tension for the PVDF-HFP and the PVDF-HFP/Zn(4-PPOx)_4_Pc membranes.

Figure 9 shows the cosine of the contact angle plotted against the surface tension for the PVDF-HFP and PVDF-HFP/Zn(4-PPOx)_4_Pc membranes. Both membranes exhibited high cosine values near 1 at low surface tensions (approximately 30 N/m), indicating high hydrophilicity and low contact angles. A sharp decline in cosine values was observed around a critical surface tension of 26.85 N/m, marking a transition to hydrophobic behavior. Beyond this point, the cosine values stabilized between −0.4 and −0.7, reflecting high contact angles and sustained hydrophobicity. While both membranes followed a similar trend, the PVDF-HFP consistently showed slightly higher cosine values than the PVDF-HFP/Zn(4-PPOx)_4_Pc, indicating that the addition of Zn(4-PPOx)_4_Pc marginally reduces the hydrophobicity of the PVDF-HFP membrane, highlighting the impact of surface tension on membrane wettability and the slight modification in hydrophobicity due to phthalocyanine incorporation.

The results of morphological studies (AFM) and contact angle trends demonstrated that the membrane’s surface roughness and texture have a significant impact on its wettability and surface energy characteristics. The rougher PVDF-HFP membrane exhibited a higher degree of hydrophobicity at higher surface tensions. Conversely, the PVDF-HFP/Zn(4-PPOx) _4_Pc membrane, with its less rough but more complex surface structure, maintains better initial wettability and a more controlled increase in hydrophobicity. This suggests that the surface modifications in the PVDF-HFP/Zn(4-PPOx) _4_Pc membrane help balance the surface energy components, leading to improved performance in managing wettability and surface tension.

These results align with the conclusions of studies by researchers such as Ridgway et al. and other works referenced in [47,48,49,50,51,52], which demonstrated that modifications in membrane surface roughness can significantly impact fouling behavior and surface energy calculations. Ridgway’s study on bacterial adhesion highlighted the critical role of surface roughness in biofilm formation, emphasizing that hydrophobic interactions between bacterial cell surface components and the membrane surface are crucial during the initial stages of adhesion and biofilm development. Furthermore, modifying the contact angles using the roughness index based on AFM measurements and the Wenzel equation, as suggested by researchers [50,51,52], provides a more accurate assessment of the surface energy parameters. This approach enhances our understanding of how surface modifications influence membrane performance in filtration and desalination applications.

### 3.3. Molecular Modeling Study

#### 3.3.1. Building Model Molecules for PVDF-HFP, Zn(4-PPOx)_4_Pc, PVDF-HFP/Zn(4-PPOx)_4_Pc

**Figure 10:**
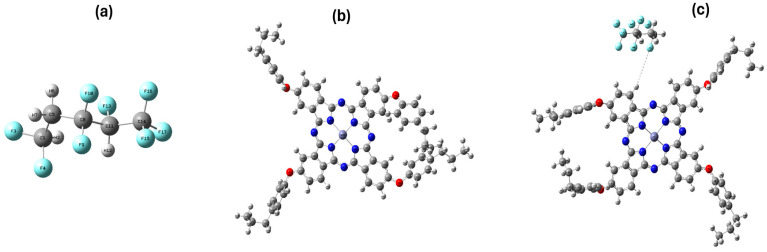
Model molecules for PVDF-HFP (**a**), Zn(4-PPOx)_4_Pc (**b**) and PVDF-HFP/Zn(4-PPOx)_4_Pc (**c**) calculated at B3LYP/LANL2DZ level. [C in grey, H in white grey, O in red, N in blue, Zn in purple and F in light blue].

The polarisability, hyperpolarizability, and dipole moment combined with these properties could contribute to the usefulness of materials that constitute reverse osmosis membranes in antibacterial applications. To calculate the electronic properties of the PVDF-HFP monomer, zinc tetra(4-propylphenoxy) phthalocyanine (Zn(4-PPOx)_4_Pc), and PVDF-HFP/zinc tetra(4-propylphenoxy) phthalocyanine composites, model structures that reflect PVDF-HFP and the interactions of Zn(4-PPOx)_4_Pc must first be developed. As indicated in Figure 10, optimization of the model system (PVDF-HFP/Zn(4-PPOx)_4_Pc) showed that the origin of the interaction was mainly due to the formation of halogen–hydrogen bonds involving the fluorine atoms of the PVDF-HFP monomer. The incorporation of Zn(4-PPOx)_4_Pc into the PVDF-HFP nanofibers by immobilization could be the origin of the improvement in the electrical and optical properties of the obtained composite, as well as its power as an antifouling in reverse osmosis processes.

#### 3.3.2. Energy Calculations

Calculations of the polarizability, hyperpolarizability, dipole moment, total energy, and their various interactions through halogen–hydrogen bonding formation were conducted for the constructed model molecules using the high-level Density Functional Theory (DFT) approach at the B3LYP/6-31 + G(d,p) levels. Table 2 compares the calculated values for the Total Energy (E), Total Dipole Moment (DM), polarisability (α), hyperpolarizability (β), and HOMO/LUMO Bandgap Energy (ΔE) for PVDF-HFP, Zn(4-PPOx)_4_Pc, and their composite PVDF-HFP/Zn(4-PPOx)_4_Pc using DFT with B3LYP/6-31 + G(d,p). PVDF-HFP is the least stable with the highest energy (−26.9864 keV) and has moderate polarisability and low hyperpolarizability, suggesting limited nonlinear optical activity. Zn(4-PPOx)_4_Pc shows greater stability (−139.954 keV), significantly higher polarizability (1137.99035 a.u.), and hyperpolarizability, indicating an enhanced response to electric fields and potential for nonlinear optical applications.

The composite has the lowest energy (−166.945 KeV), suggesting the highest stability, and exhibits the highest dipole moment, which could enhance its interaction with polar substances. Both Zn(4-PPOx)_4_Pc and the composite displayed lower bandgap energies (approximately 2 eV), indicating better conductivity and potential semiconductor applications. Consequently, the calculated physical and electronic parameters confirm the potential of PVDF-HFP to interact with Zn(4-PPOx)_4_Pc through a halogen–hydrogen bond, resulting in a highly stable and remarkably reactive structure.

#### 3.3.3. Distribution of HOMO/LUMO Molecular Orbitals in Zn(4-PPOx)_4_Pc Interacting with PVDF-HFP

**Figure 11:**
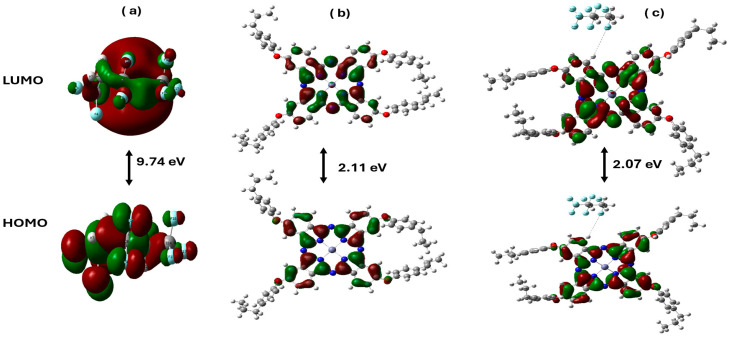
HOMO-LUMO orbital distribution with its bandgap energy (ΔE) for Model molecules for PVDF-HFP (**a**), Zn(4-PPOx)_4_Pc (**b**) and PVDF-HFP/Zn(4-PPOx)_4_Pc (**c**) calculated at B3LYP/6-31G+(d,p) level.

Figure 11 shows the distribution of the HOMO/LUMO molecular orbitals of PVDF-HFP, Zn(4-PPOx)4Pc, and their composite PVDF-HFP/Zn(4-PPOx)_4_Pc with their corresponding ΔE values. PVDF-HFP showed a large energy gap of 9.74 eV, indicating low conductivity and high stability, with tightly packed orbitals, suggesting limited reactivity. In contrast, Zn(4-PPOx)4Pc features a much smaller gap of 2.11 eV, suggesting a higher conductivity and reactivity owing to better electron delocalization. The composite material demonstrated the closest HOMO and LUMO integration with a gap of 2.07 eV, resulting from effective hydrogen and fluorine bonding between Zn(4-PPOx)4Pc and PVDF-HFP. This interaction enhances orbital delocalization across the composite, indicating the potential for tailored electrical and chemical properties suitable for advanced applications.

From these HOMO and LUMO energy values, global chemical reactivity descriptors were evaluated including chemical potential (μ), chemical hardness η, chemical softness s, and electrophilicity index (Table 3).

Table 3 compares the molecular energy parameters of PVDF-HFP, Zn(4-PPOx)_4_Pc, and their composite PVDF-HFP/Zn(4-PPOx)_4_Pc, revealing their distinct properties. PVDF-HFP demonstrated higher stability with a lower HOMO energy (−10.1931 eV) and a higher LUMO energy (−0.4571 eV), indicating lower chemical reactivity, as further evidenced by its high ionization potential (10.1931 eV) and lower electron affinity (0.4571 eV). In contrast, Zn(4-PPOx)_4_Pc and the composite exhibit lower LUMO energies and higher electron affinities, suggesting a greater propensity to accept electrons and increased reactivity. Zn(4-PPOx)_4_Pc shows significantly lower chemical hardness (1.0530) and higher softness (0.4748), indicating that it is more chemically active, a trend also observed in the composite, but to a lesser extent. The electrophilicity index of PVDF-HFP was the highest (2.4339), correlating with its lower reactivity compared to those of Zn(4-PPOx)_4_Pc and the composite. These results highlight the intermediate properties of the composite, suggesting a balance between the stability of PVDF-HFP and the reactivity of Zn(4-PPOx)_4_Pc, which could be advantageous for applications requiring controlled chemical activity.

#### 3.3.4. Molecular Electrostatic Potential (MESP)

**Figure 12:**
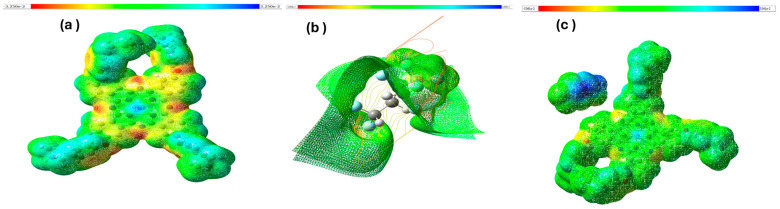
MESP maps for Zn(4-PPOx)_4_Pc (**a**), PVDF-HFP (**b**), and PVDF-HFP/Zn(4-PPOx)_4_Pc (**c**) calculated at B3LYP/6-31 G(d,p) level.

Molecular Electrostatic Potential (MESP) mapping is a potent analytical tool because it can depict the distribution and transfer of electrostatic charges across a molecule and its chemically active sites. These visualizations delineate the electron distribution within molecules and elucidate the correlation between the molecular structure and activity, including their electrophilic and nucleophilic properties. Distinct colors were utilized to identify varying electrostatic potentials, with red, blue, and green surfaces representing electrophilic, nucleophilic, and neutral regions, corresponding to negative, positive, and zero electrostatic potentials, respectively. Figure 12 shows the MESP maps of Zn(4-PPOx)_4_Pc, PVDF-HFP, and their composites. The MESP map of Zn(4-PPOx)_4_Pc (a) illustrates a complex range of electrostatic potentials, with red and blue areas indicating the electrophilic and nucleophilic sites, respectively. In contrast, the MESP map of PVDF-HFP (c) was predominantly green, signifying a more neutral electrostatic nature, which is consistent with its chemical stability. The composite material PVDF-HFP/Zn(4-PPOx)_4_Pc (b) integrates these characteristics, as demonstrated by a mesh overlay that depicts the molecular interactions between the embedded Zn(4-PPOx)_4_Pc and the PVDF-HFP matrix, highlighting the intricate interplay at the molecular level.

### 3.4. Molecular Docking Studies

The comparison of the proteins from *S. aureus*, *P. aeruginosa*, and *E. coli* in terms of their interactions with Zn(4-PPOx)_4_Pc highlights significant differences in binding affinities and inhibitory effects. The interaction data and best docking pose of [Zn(4-PPOx)_4_Pc]-(4XO8), [Zn(4-PPOx)_4_Pc]-(3PBQ) and [Zn(4-PPOx)_4_Pc]-(1N67) are displayed in Table 4.

**Figure 13:**
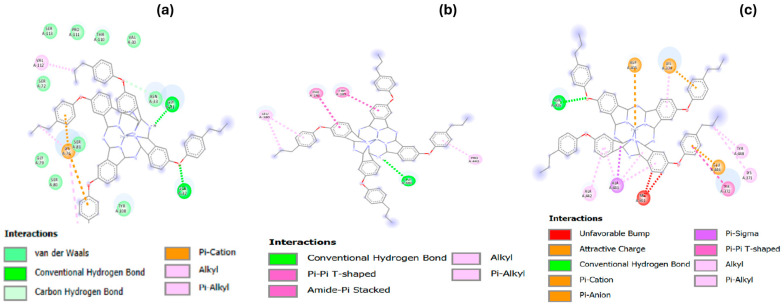
Two-dimensional illustration of interactions of (**a**) *E. coli* (4XO8)-[PPPcZn]—(**b**) *P. aeruginosa* (3PBQ) [PPPcZn]—(**c**) *S. aureus* (1N67)-[PPPcZn].

The protein from *S. aureus* (1N67) showed the highest binding affinity, reflected by a binding energy of -8.56 kcal/mol. This strong affinity was correlated with its low inhibition constant of 528.94 nM, demonstrating a potent inhibitory effect. The robust binding affinity can be attributed to the significant electrostatic interactions (with an electrostatic energy of −10.16 kcal/mol) within the binding sites with residues through π-cation (LYS374) and π-anion (GLU446) bonds. In addition, Zn(4-PPOx)_4_Pc provided a strong and stable interaction with various hydrophobic interactions with alkyl LYS371, aromatic residues (TYR372, TYR448, LYS374, and ALA442), and the presence of a hydrogen bond (ASN406). Collectively, these factors reinforce the effective binding of the protein and contribute to the strong inhibitory effect of Zn(4-PPOx)_4_Pc against *S. aureus* (Figure 13). In contrast, the protein from *P. aeruginosa* (3PBQ) showed a moderate binding affinity of −5.48 kcal/mol, accompanied by a higher inhibition constant (95.67 µM). This weaker binding affinity was partly due to the lack of significant electrostatic interactions. The simple interaction profile of this protein suggests that Zn(4-PPOx)_4_Pc does not bind effectively, leading to a less potent inhibitory effect. Meanwhile, the protein from *E. coli* (4XO8) exhibits the weakest binding affinity, with a binding energy of −3.05 kcal/mol and the highest inhibition constant of 5.77 mM. The absence of electrostatic interactions limited the ability of the protein to interact strongly with PPPcZn, resulting in a markedly reduced inhibitory effect. Figure 13 displays 2D illustrations of the interactions between the ligand and receptor. Other details (name, distance, category, and type) of the interactions are illustrated in Table 4.

Overall, these findings from a molecular docking simulation emphasize that the predicted structural and chemical properties of the *S. aureus* protein enable it to form more complex and diverse interactions with Zn(4-PPOx)_4_Pc, making it highly susceptible to inhibition. In contrast, the simpler binding interactions of *P. aeruginosa* and *E. coli* proteins lead to significantly reduced binding affinities and, thus, weaker inhibitory effects. The information provided can help in understanding the binding affinity and other energetics related to the interaction between a Zn(4-PPOx)_4_Pc as a ligand and its target. This result corroborates the findings that metallophthalocyanines have a strong affinity for proteins from *S. aureus*, leading to a potent inhibitory effect, while their affinity for proteins from *P. aeruginosa* and *E. coli* is comparatively lower, resulting in weaker inhibition [54,55].

#### Hydrophobicity, Aromatic Surfaces, Hydrogen Bonds, Ionizability, and Solvent-Accessible Surface

**Figure 14:**
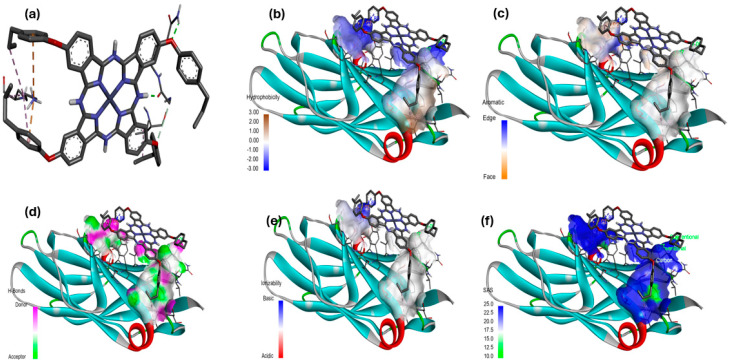
Molecular docking data of *E. coli* (4XO8)-[Zn(4-PPOx)_4_Pc] complex; (**a**) specific interaction of Zn(4-PPOx)_4_Pc; (**b**) hydrophobic surfaces; (**c**) aromatic surface, (**d**) bonding surface of hydrogen, (**e**) ionizability, and (**f**) solvent-accessible surface (SAS).

**Figure 15:**
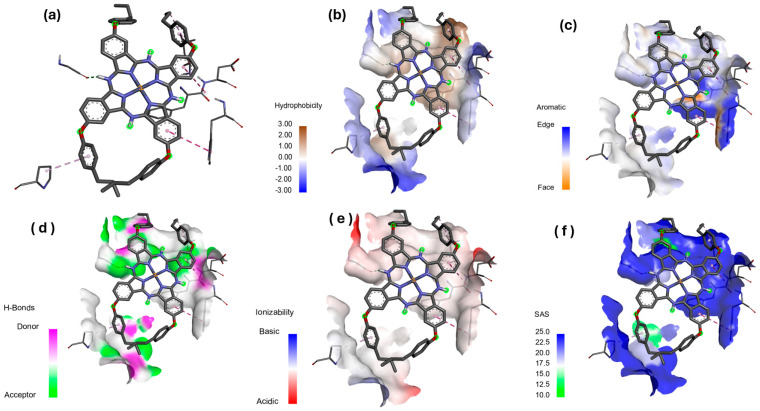
Molecular docking data of *P. aeruginosa* (3PBQ)-[Zn(4-PPOx)_4_Pc] complex; (**a**) Specific interaction of Zn(4-PPOx)_4_Pc, (**b**) hydrophobic surfaces; (**c**) aromatic surface, (**d**) bonding surface of hydrogen, (**e**) ionizability, and (**f**) solvent-accessible surface (SAS).

**Figure 16:**
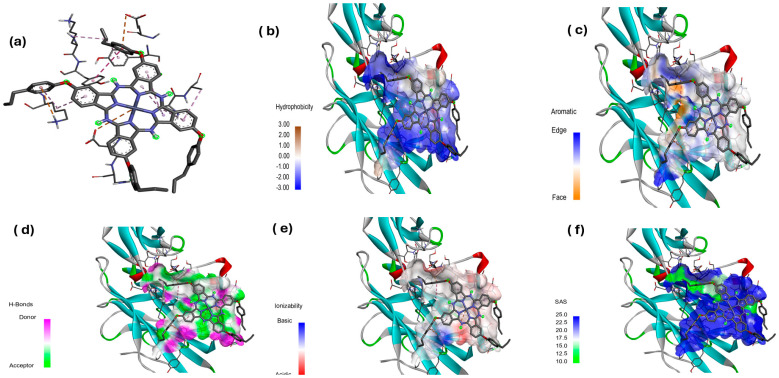
Molecular docking data of *S. aureus* (1N67)-[Zn(4-PPOx)_4_Pc]; (**a**) specific interaction of Zn(4-PPOx)_4_Pc; (**b**) hydrophobic surfaces; (**c**) aromatic surface, (**d**) bonding surface of hydrogen, (**e**) ionizability, and (**f**) solvent-accessible surface (SAS).

Ligand-receptor complexes were analyzed using the Discovery Studio Visualizer (DSv) 2.5 software package (Accelyys, San Diego, CA, USA). The molecular docking results of the [Zn(4-PPOx)_4_Pc] complex with proteins from *E. coli* (4XO8), *P. aeruginosa* (3PBQ), and *S. aureus* (1N67) are displayed in Figure 14, Figure 15 and Figure 16, respectively. Each figure presents six panels illustrating the specific interactions of [Zn(4-PPOx)_4_Pc] with the protein (a), hydrophobic surfaces (b), aromatic surfaces (c), hydrogen bonding surfaces (d), ionizability (e), and solvent-accessible surface (SAS) (f). The comparison of these three figures indicates differences in how [Zn(4-PPOx)_4_Pc] interacts with each bacterial protein, suggesting variations in their binding efficiencies and biological effects, which are crucial for developing targeted antimicrobial strategies.

## 4. Conclusions

In this study, zinc 2,9,16,23-tetra(4-propylphenoxy) phthalocyanine (Zn(4-PPOx) _4_Pc) was successfully synthesized and integrated into PVDF-HFP membranes to develop a novel nanocomposite with potential applications for reducing biofouling in water desalination processes. Structural and chemical characterizations confirmed the presence of Zn(4-PPOx) _4_Pc within the PVDF-HFP matrix, as evidenced by FTIR and UV-Vis spectroscopy. AFM analysis revealed that the inclusion of Zn(4-PPOx) _4_Pc resulted in a smoother membrane surface, which was beneficial for minimizing fouling. The wettability tests indicated that the Zn(4-PPOx) _4_Pc-modified membranes exhibited slightly reduced hydrophobicity compared to the unmodified PVDF-HFP membranes, which could enhance the antifouling performance. DFT computational studies provided a deeper understanding of the interactions between PVDF-HFP and Zn(4-PPOx) Pc, thereby demonstrating the improved stability and reactivity of the composite material. Molecular docking studies further demonstrated the strong binding affinity of Zn(4-PPOx) _4_Pc to bacterial proteins, particularly those from *S. aureus*, suggesting its potential antimicrobial benefits. These findings highlight the promising potential of Zn(4-PPOx) _4_Pc/PVDF-HFP nanocomposite membranes in improving the efficiency of water desalination by reducing fouling and providing antibacterial properties. Future work will focus on further optimizing the membrane fabrication process and evaluating the long-term performance and scalability of these nanocomposite membranes for real-world desalination applications.

## Figures and Tables

**Table 1:** Comparison of the roughness parameters between the PVDF-HFP/Zn(4-PPOx) _4_Pc membrane and the PVDF-HFP control membrane.

Membranes		
	PVDF-HFP/Zn(4-PPOx)_4_Pc	PVDF-HFP
Area Roughness		
Area	2.51 nm^2^	2.52 nm^2^
Average Roughness (Sa)	651.58 nm	763.44 nm
Root Mean Square Roughness (Sq)	817.57 nm	938.73 nm
Maximum Height of Surface (Sy)	6.2243 µm	5.6323 µm
Maximum Peak Height (Sp)	3153.8 nm	2702.7 nm
Maximum Valley Depth (Sv)	−3070.5 nm	−2929.6 nm
Mean Roughness Depth (Sm)	−18.458 fm	−17.94 fm
Line Roughness		
Average Roughness (Ra)	686.04 nm	786.6 nm
Root Mean Square Roughness (Rq)	807.47 nm	980.08 nm
Maximum Height (Ry)	3.1701 µm	4.4474 µm
Maximum Peak Height (Rp)	1764.5 nm	2217.9 nm
Maximum Valley Depth (Rv)	−1405.6 nm	−2229.5 nm
Mean Roughness Depth (Rm)	−18.626 fm	−18.554 fm

**Table 2:** Total energy (E) in KeV, Total Dipole Moment (DM) in Debye, Polarisability α(Pol α) in a.u., Hyperpolarisability β (HyPol β) in a.u and HOMO/LUMO Bandgap Energy (ΔE) in eV for PVDF-HFP, Zn(4-PPOx)4Pc, and PVDF-HFP/Zn(4-PPOx)_4_Pc were calculated Using DFT: B3LYP/6-31 + G(d,p).

Structure	E (KeV)	DM (Debye)	Pol α (a.u.)	HyPol β (a.u.)	ΔE (eV)
PVDF-HFP	−26.9864	1.36969	65.32976	2.567644	9.74
Zn(4-PPOx)_4_Pc	−139.954	1.37243	1137.99035	1804.66747	2.11
PVDF-HFP/Zn(4-PPOx)_4_Pc	−166.945	4.55515	nc	nc	2.07

nc: not calculated.

**Table 3:** Calculated global chemical reactivity descriptors [53].

Molecules	PVDF-HFP	Zn(4-PPOx)_4_Pc	PVDF-HFP/Zn(4-PPOx)_4_Pc
E_LUMO_	−0.457151654	−2.732025358	−3.025364336
E_HOMO_	−10.19312131	−4.838188334	−5.098601508
Ionization potential (IP = −E_HOMO_)	10.19312131	4.838188334	5.098601508
Electron affinity (EA = −E_LUMO_)	0.457151654	2.732025358	3.025364336
Chemical hardness (η = (IP − EA)/2)	4.867984826	1.053081488	1.036618586
Chemical softness (s = 1/2η)	0.102711906	0.474797065	0.482337483
Chemical potential (μ = (IP − EA)/2)	4.867984826	1.053081488	1.036618586
Electronegativity (χ = (1 + EA)/2)	0.728575827	1.866012679	2.012682168
Electrophilicity index (ω = μ^2^/2η)	2.433992413	0.526540744	0.518309293

**Table 4:** Binding affinity (kcal/mol) and nonbonding interactions of Zn(4-PPOx)_4_Pc, within the active site of the bacterial proteins *E. coli* (PDB ID: 4XO8), *S. aureus* (PDB ID: 1N67), and *P. aeruginosa* (PDB ID: 3PBQ).

Entry	Protein	Binding Affinity (kcal/mol)	Bond Category	Residues in Contact	Interaction Types	Distance ($\overset{\cdot }{Å}$)
1	4XO8	−3.05	Hydrogen	GLN32	H	2.06173
			Hydrogen	GLY31	CH	3.53301
			Electrostatic	LYS76	PiCa	4.87757
			Electrostatic	LYS76	PiCa	2.91888
			Hydrophobic	VAL112	Pi-A	5.14891
			Hydrogen	GLN32	H	2.06173
			Hydrogen	GLY31	H	3.53301
2	3PBQ	−5.48	Hydrogen	GLY380	H	2.47192
			Hydrophobic	TRP185	PiPiTS	5.07933
			Hydrophobic	PHE182	AmPiS	5.14985
			Hydrophobic	LEU180	A	4.60392
			Hydrophobic	LEU180	PiA	5.10146
			Hydrophobic	PRO443	PiA	4.38758
3	1N67	−8.56	Electrostatic	ASP405	AtCh	5.35046
			Hydrogen	ASN406	H	2.27904
			Electrostatic	LYS374	PiCa	4.76248
			Electrostatic	GLU446	PiAn	4.65720
			Hydrophobic	ALA441	PiS	3.55853
			Hydrophobic	TYR372	PiPi TS	5.58462
			Hydrophobic	LYS371	Alkyl	4.70072
			Hydrophobic	TYR448	PiA	4.29719
			Hydrophobic	LYS374	PiA	4.81324
			Hydrophobic	ALA442	PiA	4.36383
			Hydrophobic	ALA442	PiA	4.7961
			Hydrophobic	ALA442	PiA	4.22284
			Hydrophobic	ALA442	PiA	5.2594

H = Conventional hydrogen bond; CH = Carbon hydrogen bond; A = Alkyl; PiA = Pi-alkyl; PiAn = Pi = An; PiCa = Pi-cation; PiS = Pi-sigma; AtCh = Attraction charge; Pi-S = Pi-sigma; PiPi = Pi-Pi; PiPiTS = Pi-Pi T-shaped; AmPiS = Amide-Pi S stacked.

## Data Availability

Data are contained within the article.
